# *In Vivo* Predictive Dissolution Testing of Montelukast Sodium Formulations Administered with Drinks and Soft Foods to Infants

**DOI:** 10.1208/s12249-020-01825-7

**Published:** 2020-10-13

**Authors:** J. Martir, T. Flanagan, J. Mann, N. Fotaki

**Affiliations:** 1 Department of Pharmacy and Pharmacology, University of Bath, Claverton Down, Bath, BA2 7AY UK; 2 Oral Product Development, Pharmaceutical Technology & Development, Operations, AstraZeneca, Macclesfield, UK; 3 UCB Pharma, Chemin du Foriest, B - 1420 Braine-l’Alleud, Belgium

**Keywords:** paediatrics, drug manipulation, food, *in vitro* dissolution, mini-paddle, *in vitro-in vivo* relationship, paediatric biorelevant media

## Abstract

*In vitro* dissolution testing conditions that reflect and predict *in vivo* drug product performance are advantageous, especially for the development of paediatric medicines, as clinical testing in this population is hindered by ethical and technical considerations. The aim of this study was to develop an *in vivo* predictive dissolution test in order to investigate the impact of medicine co-administration with soft food and drinks on the dissolution performance of a poorly soluble compound. Relevant *in vitro* dissolution conditions simulating the *in vivo* gastrointestinal environment of infants were used to establish *in vitro*-*in vivo* relationships with corresponding *in vivo* data. Dissolution studies of montelukast formulations were conducted with mini-paddle apparatus on a two-stage approach: infant fasted-state simulated gastric fluid (Pi-FaSSGF; for 1 h) followed by either infant fasted-state or infant fed-state simulated intestinal fluid (FaSSIF-V2 or Pi-FeSSIF, respectively; for 3 h). The dosing scenarios tested reflected *in vivo* paediatric administration practices: (i.) direct administration of formulation; (ii.) formulation co-administered with vehicles (formula, milk or applesauce). Drug dissolution was significantly affected by co-administration of the formulation with vehicles compared with after direct administration of the formulation. Montelukast dissolution from the granules was significantly higher under fed-state simulated intestinal conditions in comparison with the fasted state and was predictive of the *in vivo* performance when the granules are co-administered with milk. This study supports the potential utility of the *in vitro* biorelevant dissolution approach proposed to predict *in vivo* formulation performance after co-administration with vehicles, in the paediatric population.

## INTRODUCTION

Understanding the dissolution profile of a pharmaceutical dosage form and linking it to its *in vivo* pharmacokinetic (PK) profile is a vital requirement for ensuring product quality and safety of use (1–3). Dissolution profiles can be analysed through different approaches: using model-dependent methods where experimental data are fitted using mathematical equations, model-independent methods (single values such as mean dissolution time and area under the dissolution curve (AUC) are used for data evaluation) and/or statistical methods (*e.g.* ANOVA and multivariate analysis) (4,5).

Drug dissolution profiles may be used to establish *in vitro*-*in vivo* correlations (IVIVC). The development of an IVIVC for a pharmaceutical dosage form is of great interest to the pharmaceutical industry and plays a key role in the pharmaceutical development of dosage forms (1). It can serve as a surrogate for *in vivo* bioavailability and be used to request biowaiver status for formulations or production changes within a product lifecycle (1–3). This reduces the need for expensive bioequivalence (BE) testing in humans.

Defining appropriate biorelevant drug dissolution conditions requires an understanding of the relationship between the various physicochemical and physiological factors that have an impact on the rate and extent to which an orally administered dosage form is absorbed (4). Since clinical research with adults cannot simply be generalised or extrapolated to the paediatric population, research involving paediatric patients is essential (6). Age-related PK studies are normally required due to differences in anatomy or drug handling/manipulation practices, which might lead to different dose requirements to achieve efficacy or to avoid adverse effects (7). Moreover, changes in developmental physiology throughout childhood complicate pharmacotherapy, due to its impact on drug absorption, distribution, metabolism and excretion of drugs and excipients (8). Thus, better understanding of physiological developmental changes is important for paediatric drug testing. Data characterising the GI environment of the different paediatric age groups is very difficult to be obtained due to ethical constraints, associated co-morbidities in paediatric patients and the need for parental consent. Challenges in paediatric medicine development further include (i.) the need for appropriate outcome measures for paediatric patients, (ii.) the complexities of paediatric administration practices (*e.g.* drug manipulation and mixing with food and drinks (vehicles)), (iii.) the parental involvement and (iv.) the adaptations of required research procedures and settings to accommodate paediatric anatomic/cognitive development (8).

Development of a physiologically relevant *in vitro* dissolution setup would be crucial for the prediction of the *in vivo* performance after the administration of a formulation to a paediatric patient. Moreover, it would be beneficial for the investigation of formulation sensitivity to different foods and drinks, so that the risks associated with its co-administration can be predicted. In 2018, the FDA issued a draft guidance addressing the recommended approaches for determination of the suitability of the vehicles intended for co-administration of paediatric medicines. In this guidance, standardised *in vitro* methods for evaluating possible vehicle effects on *in vivo* product performance were described (9). These tests could help reduce the number of *in vivo* studies required for paediatric formulation development, and ultimately help tackle ethical issues related to paediatric clinical research (10). To this extent, *in vitro* test conditions should address the parameters relevant to drug release and dissolution in the paediatric gastrointestinal (GI) tract, including media composition, prandial state, hydrodynamics and current administration practices. The possible effect of these parameters on the *in vivo* drug behaviour should be considered during paediatric drug development (6,11). Recently, *in vitro* dissolution studies, performed with a mini-paddle apparatus and a two-stage approach, showed that this setup could be a useful biopharmaceutical tool for estimating drug release/dissolution in paediatric conditions (12,13). With this setup, it is possible to address pH, fluid volumes and transit times representative of the GI tract of infants, as well as different paediatric administration practices such as medicine co-administration with food and drinks.

The aims of this study were (i.) to investigate the impact of co-administration of montelukast formulations (granules and chewable tablets) with food and drinks on drug dissolution performance, under paediatric physiological relevant conditions, and (ii.) to evaluate the *in vitro* dissolution studies in terms of their predictability of the *in vivo* formulation performance.

Montelukast was chosen as the model drug; it is an amphoteric compound, with high lipophilicity (clogP 8.79), and classified as a BCS class II compound (14). Montelukast is a potent leukotriene receptor antagonist that has demonstrated efficacy and tolerability in the treatment of patients with chronic asthma (15–17). For approved paediatric use, it is available in two dosage forms (granules and chewable tablets), and is used in very young ages from 1 month old (17). The PK profile of montelukast is dose proportional and not substantially altered by age (18). As shown in different *in vivo* studies in infant subgroups, montelukast formulations are often mixed with drinks or soft foods to facilitate administration (7,16,17,19).

## MATERIALS AND METHODS

### Materials

Ammonium acetate (high-performance liquid chromatography (HPLC) grade), 37% hydrochloric acid, sodium hydroxide, sodium chloride, glacial acetic acid and maleic acid were purchased from Fisher Scientific (UK). Dichloromethane, acetonitrile (HPLC grade) and methanol (HPLC grade) were from VWR Chemicals (UK). Montelukast sodium (pharmaceutical secondary standard), sodium oleate and pepsin from porcine gastric mucosa (Ph. Eur.) were obtained from Sigma-Aldrich Company Ltd. (UK). Sodium taurocholate (Prodotti Chimici Alimentari S.P.A., Italy), egg lecithin Lipoid EPCS (Lipoid E PCS, Phosphatidylcholine from egg; from Lipoid GmbH, Germany) and glyceryl monooleate – Rylo Mg 19 (Danisco, Denmark) were used. Water was ultra-pure (Milli-Q) laboratory grade. Regenerated cellulose (RC) membrane filters (0.45 μm) (Cronus®, UK), filter papers (0.45 μm) and glass microfiber (GF/D) filters (2.7 μm) (Whatman®, UK) and porous full flow polyethylene cannula filters (10 μm) (Quality Lab Accessories LCC, USA) were used. Full fat U.H.T. milk was purchased from The Co-Operative (UK), and first infant milk (cow’s milk-based formula) was from Cow & Gate (UK) and Bramley applesauce Colman’s of Norwich from Unilever (UK). Singulair^®^ Paediatric granules (4 mg, 28 sachets; from Merck Sharp & Dohme Ltd., UK) and Actavis^®^ chewable tablets (5 mg, 28 chewable tablets; from Actavis, UK) were kindly donated by AstraZeneca (UK).

### Methods

#### Dissolution Media Preparation

Paediatric biorelevant media representative of infants were freshly prepared for each experiment, as described by Maharaj *et al.* (20). Infant fasted-state simulated gastric fluid (Pi-FaSSGF, pH 1.6) and fasted-state simulated intestinal fluid (FaSSIF-V2, pH 6.5) or infant fed-state simulated intestinal fluid (Pi-FeSSIF, pH 5.8) were used. Both fasted and fed intestinal state were simulated since the prandial state of the infant patients in the *in vivo* studies was not reported, and in order to investigate if medicine co-administration with a vehicle would induce a food effect in the infant. Double concentrated simulated intestinal fluids were prepared for the two-stage dissolution studies performed (“Biorelevant *In Vitro* Dissolution Studies”).

#### Sample Preparation

Formula was prepared as per manufacturer’s instructions: 1 scoop of powder (approximately 4.5 g) was added to 30 mL of boiled cooled water. Two formulations were tested: Singulair^®^ granules (4 mg) and Actavis^®^ chewable tablets (5 mg) which were crushed prior to mixing (following reported practices (7)). For the direct administration scenario, formulations were tested in the simulated GI fluids without prior mixing with a vehicle. For the mixing with vehicle scenario, each sample was prepared by addition of the formulation to milk (25 mL; as previously investigated (12)), applesauce (15 g) or formula (5 mL), followed by mixing with a stainless-steel spatula. Mixing with formula was performed only for the Singulair^®^ granules to mimic the *in vivo* study dosing scenario (17). The preparation technique procedure was time-controlled (less than 2 min were spent between preparation and dosing of the mixture), and the mixing was performed in exactly 30 s.

#### Biorelevant *In Vitro* Dissolution Studies

Dissolution studies were performed with a mini-paddle apparatus (Agilent Technologies 708-DS apparatus configured with TruAlign 200 mL vessels and electropolished stainless-steel mini-paddles; Agilent, USA). Experiments were conducted at 37°C, and agitation rate was set to 50 revolutions per minute (rpm). A two-stage approach was followed: gastric conditions were simulated for 1 h (Pi-FaSSGF pH 1.6; total volume with sample: 100 mL), followed by intestinal simulated conditions (FaSSIF-V2 pH 6.5 or Pi-FeSSIF pH 5.8; final volume: 200 mL), for 3 h. Sample collection took place at 5, 15, 30, 45, 60, 75, 90, 120, 180 and 240 min. Samples of 2 mL were withdrawn (with volume replacement with the corresponding media), using a 2-mL glass syringe (Fortuna Optima^®^ fitted with a stainless tubing) through a cannula fitted with a full flow filter (10 μm). All experiments were performed without direct light exposure to avoid photodegradation of montelukast (21). After collection, samples were filtered through a GF/D filter (2.7 μm), treated, placed into amber HPLC vials and injected into the HPLC. Treatment was as follows: 1000 μL of acetonitrile was added to 500 μL of the filtered sample, the mixture was vortexed (HTZ, UK) for 1 min and centrifuged (8000 rpm, 15 min, 4°C) (Beckman Coulter J2-MC centrifuge, UK) and the supernatant was filtered through a RC filter (0.45 μm). The pH of the media was measured at the end of each experiment to ensure the pH shift had been successful and that the vehicle did not alter the media pH.

The effect of different administration scenarios on drug dissolution was investigated by varying the mode of the introduction of the formulation in the simulated gastric fluid in the dissolution vessel: direct administration of the formulation or administration of the formulation after mixing with drinks (formula and milk) or soft food (applesauce). These vehicles were selected based on their impact on the dissolution of montelukast (12) and/or to mimic the *in vivo* studies performed in infants (12,16,17,19). The composition and physicochemical properties of these vehicles, including pH, buffer capacity and viscosity, have been recently published and discussed (13).

All experiments were performed in triplicate. Fresh calibration curves (concentration range: 0.5–60 μg/mL) were prepared in the corresponding media, by appropriate dilution of a 1000 μg/mL stock solution of montelukast analytical standard in methanol. Results were expressed as mean percentage (%) drug dissolved ± standard deviation (S.D.) at the given sampling time.

#### Chromatographic Conditions for Drug Analysis

The chromatographic method used for quantification of montelukast was a modification of the method by Raju *et al.* (22). Drug quantification was performed with HPLC with ultraviolet (UV) detection (Agilent HPLC system 1100/1200 series; Agilent, USA), using a C_18_ column (RP Agilent Eclipse XDB, 250 mm × 4.6 mm, 5 μm particle size), and ammonium acetate buffer pH 5.5 (A) and methanol (B) as mobile phase, delivered on a linear gradient. The selected gradient started with 10% of solvent B, which was increased to 50% within 2 min, and 90% within 4 min; at 11.30 min, the initial conditions of analysis were re-established. Injection volume was 100 μL, flow rate was 1 mL min^−1^, run time was 12.30 min, detection wavelength was 284 nm and column temperature was 20°C.

#### Data Analysis

##### *In Vitro* Data Analysis

The linear trapezoidal method was used to calculate the area under the curve of each *in vitro* % drug dissolved over 4-h profile (AUC_0–4 h_ *in vitro*). One-way analysis of variance (ANOVA) with a post hoc Tukey honest significant difference (HSD) test was conducted to investigate differences in the AUC_0–4 h_ *in vitro* calculated from the dissolution studies, after direct administration of formulation and after mixing the formulation with the different vehicles (*p* < 0.05 noting significance level). *T* test analysis was used to compare experimental results under fasted-state gastric conditions, followed by fasted- or fed-state intestinal conditions (represented as Pi-FaSSGF/FASSIF-V2 and Pi-FaSSGF/Pi-FeSSIF, respectively) (significance *p* < 0.05). Analyses were performed with GraphPad Prism^®^ v.7 software (USA).

##### *In Vivo* Data Analysis

Published data of plasma concentration profiles of Singulair^®^ granules (4 mg) co-administered with formula or applesauce to different infant subgroups (formula: 1 to 3 months; applesauce: 3 to 6 months and 6 to 24 months) were digitalised with WebPlotDigitizer^®^ v4.1 software (USA) (16,17,19,23).

The corresponding *in vivo* drug absorption profiles were obtained after deconvolution of the oral data using the Wagner-Nelson equation (Eq. 1) (Excel, Microsoft®) (24).

1 $$\%\mathrm{absorbed}=\frac{A(t)}{A\left(\infty \right)}\times 100=\frac{A(t)+k\ {\int}_{\tau =0}^tA\left(\tau \right) d\tau}{k\ {\int}_{\tau =0}^{\infty }A\left(\tau \right) d\tau}\times 100$$

where *A*(*t*) is the amount of drug in the system at time *t* and *k* is the first-order elimination rate constant (24). The elimination rate constant was obtained from the slope of the terminal logarithmic concentrations of the *in vivo* montelukast oral data.

The linear trapezoidal method was used to calculate the area under the curve of each *in vivo* % drug absorbed over 4-h profile (AUC_0–4 h_ *in vivo*).

##### *In Vitro/In Vivo* Relationship

An *in vitro*-*in vivo* relationship for Singulair^®^ granules (4 mg) was investigated by comparing the *in vitro* dissolution (AUC_0–4 h_ *in vitro*) and the *in vivo* absorption data (AUC_0–4 h_ *in vivo*). Average differences between the obtained AUC_0–4 h_ *in vitro* and the AUC_0–4 h_ *in vivo* of the different subgroups were expressed as a ratio % (AUC_0–4 h_ *in vitro*/AUC_0–4 h_ *in vivo* × 100). For evaluation of the results, ratios lower than 100% indicate that AUC_0–4 h_ *in vitro* was lower than the AUC_0–4 h_ *in vivo* and higher values represent the opposite. To denote relevant discrepancies between the AUC_0–4 h_ *in vitro* and AUC_0–4 h_ *in vivo*, reference points corresponding to ratios of 80 and 125% were used. Mean ratios falling inside this reference range represent an *in vitro*-*in vivo* relationship, with *in vitro* dissolution results being predictive of the *in vivo* drug performance.

## RESULTS AND DISCUSSION

### *In Vitro* Biorelevant Drug Dissolution Studies for the Assessment of the Impact of Medicine Co-administration with Food and Drinks

**Fig. 1:**
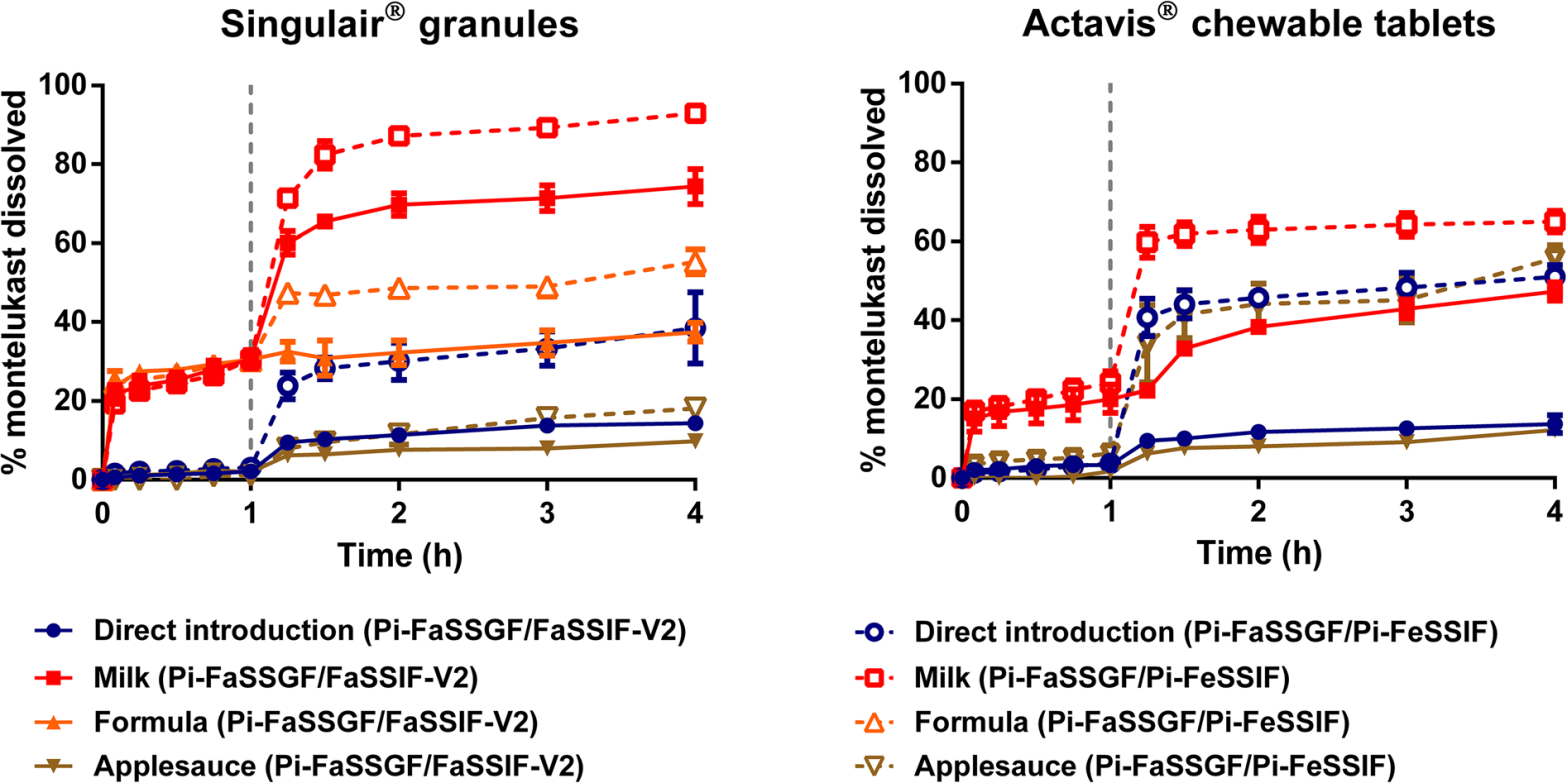
Mean % montelukast dissolved (± S.D.) from Singulair^®^ granules and Actavis^®^ chewable tablets after direct introduction of the formulation and mixing with selected vehicles, under fasted-state gastric conditions, followed by fasted-state (full lines) or fed-state (dashed lines) intestinal conditions (dotted vertical lines represent the time of medium change)

Dissolution of montelukast from both formulations in the administration scenarios tested is presented in Fig. 1. In gastric conditions (Pi-FaSSGF), dissolution of montelukast was higher when the formulations were mixed with milk and formula (for the case of Singulair^®^ granules), in comparison with direct administration and after mixing with applesauce. It should be noted that the preparation technique of the vehicle-drug mixture was controlled; therefore, it can be concluded that the differences observed are not related to the dosing preparation technique. In intestinal conditions, differences in drug dissolution were observed for both formulations under fasted- or fed-state simulated conditions (FaSSIF-V2 pH 6.5 or Pi-FeSSIF pH 5.8). These differences are probably attributed to an increase in drug solubilisation (drug solubility = 8 μg/mL and 53 μg/mL in FaSSIF-V2 and Pi-FeSSIF, respectively) due to the presence of a higher concentration of bile salts and lecithin in the fed-state simulated intestinal fluid, as shown in solubility studies of montelukast in different paediatric media (14,20,25). In addition, the vehicle impact on drug dissolution varied depending on the vehicles used for co-administration and the formulation tested. For example, when both formulations were mixed with applesauce, the impact of testing under fed intestinal conditions was higher for the crushed chewable tablets than for the granules.

**Fig. 2:**
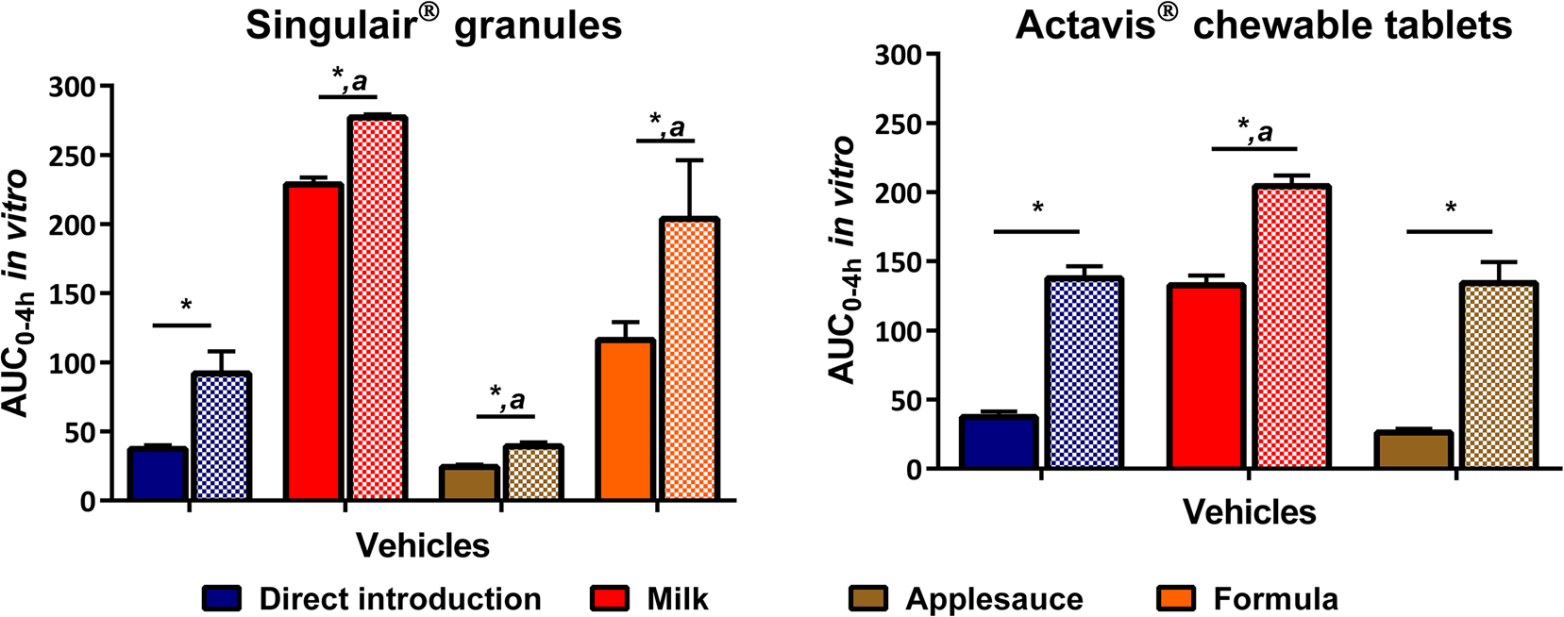
AUC_0–4 h_ *in vitro* (% dissolved * h) of montelukast dissolution profiles after direct administration of formulation (left panel: Singulair^®^ granules, right panel: Actavis^®^ chewable tablets) and after mixing with the vehicles. (*a* denotes statistical difference between direct administration (blue bars) and co-administration with vehicles (other colours); * denotes a statistical difference on drug dissolution between testing under fasted-state gastric conditions followed by fasted-state (full bar) or fed-state (dashed bar) intestinal conditions (*p* < 0.05))

Comparison of the AUC_0–4 h_ *in vitro* of the dissolution profiles (4 h) is presented in Fig. 2. Results of the AUC_0–4 h_ *in vitro* confirmed that dissolution of montelukast from the two formulations tested was significantly affected by co-administration with vehicles, compared with the direct administration scenario. The AUC_0–4 h_ *in vitro* was also shown to be significantly higher when testing under fed-state simulated intestinal conditions in comparison with the fasted state.

For Singulair^®^ granules, the AUC_0–4 h_ *in vitro* of the direct administration of formulation profile was significantly lower compared with the ones of the co-administration with drinks profiles (milk and formula), and significantly higher than the one of the co-administration with applesauce profile. Drug dissolution (4 h) was higher when the formulation was co-administered with milk (74.3 and 93.0% drug dissolved in Pi-FaSSGF/FaSSIF-V2 and Pi-FaSSGF/Pi-FeSSIF, respectively), than when mixed with formula (% drug dissolved = 37.4 (Pi-FaSSGF/FaSSIF-V2) and 55.3 (Pi-FaSSGF/Pi-FeSSIF)). These results confirm that vehicles of the same subtype (*i.e.* dairy drinks) can have different effects on drug dissolution, in accordance with what was observed in previous studies (12,13,26). The lower dissolution of montelukast observed when the granules were mixed with formula, compared with the drug dissolution when mixed with milk, relates to the differences in the solubility of montelukast in the two vehicles (milk: 13.3 mg/mL; formula: 12.0 mg/mL) (13). It can be hypothesised that these differences were accentuated by the use of different volumes of the two drinks mixed with the formulation (15 mL milk and 5 mL formula). This is of particular importance considering that the recommendations for medicine co-administration with drinks/soft foods often do not specify the volume of vehicle to use (7). These results indicate the risk of unspecific recommendations for vehicle choice, and further confirm the importance of the FDA draft guidance on vehicle selection and *in vitro* methods for product quality assessment (9). The lowest % drug dissolution (4 h) was observed for the mixing with applesauce scenario (% drug dissolved = 9.8 (Pi-FaSSGF/FaSSIF-V2) and 18.1 (Pi-FaSSGF/Pi-FeSSIF)). The solubility of montelukast in this vehicle is 5.3 mg/mL, and the pH of the vehicle is ~ 3, which might partly have contributed to the lower drug dissolution observed when the granules were mixed with applesauce, in comparison with when the formulation was mixed with formula and milk. However, the lower % drug dissolution is likely more related to the presence of starch in the composition of applesauce, which forms a net gel around the formulation and negatively affects drug release and dissolution (27). During the dosing preparation and dissolution testing, it was observed that the applesauce-granules mixture was more viscous than the formula/milk-drug mixtures. While the mixture would eventually slowly disperse once added to the media, it is not possible to distinguish the formulation from the vehicle mixture during the *in vitro* study.

The AUC_0–4 h_ *in vitro* of the crushed Actavis^®^ chewable tablets mixed with applesauce profile was not significantly different from the one of the direct introduction profile, whereas a higher AUC_0–4 h_ *in vitro* was observed after mixing with milk (Fig. 2). The higher drug dissolution when the formulation was mixed with milk is probably related to the higher drug solubilisation in milk, due to the high drug affinity for the protein and fat globules in milk, as well as the higher pH and buffer capacity of this vehicle (13,25).

Overall, it was observed that co-administration with food and drink vehicles significantly affects the dissolution of montelukast from both formulations. Results showed the influence of drug ionisation and solubility (*e.g.* higher % montelukast dissolved when formulations were mixed with milk), vehicle viscosity (*e.g.* higher viscosity of applesauce hinders the dissolution of the Singulair^®^ granules) and alteration of formulation factors (granules and crushed chewable tablets displayed different dissolution behaviours when mixed with applesauce), on drug dissolution behaviour. In addition, simulated intestinal prandial conditions were shown to affect drug dissolution behaviour, with higher % drug dissolved (4 h) achieved when testing under fed-state simulated intestinal conditions. These results indicate that the impact of the practice of medicine co-administration with food and drinks will be higher if the vehicle used triggers a food effect *in vivo* or if medicine co-administration with vehicles is performed under fed-state conditions.

### *In Vivo* Drug Absorption

In the *in vivo* studies of Singulair^®^ granules (4 mg) administered to infant patients, medicine administration was conducted by mixing the formulation with different vehicles: formula (5 mL) to infants 1 to 3 months, and applesauce (15 g) to infants from 3 to 24 months (two subgroups: 3 to 6 and 6 to 24 months). The prandial state of the patients in these studies was not disclosed and potential vehicle-induced differences on drug behaviour were not considered (16,17,19,23).

PK parameters of montelukast (*C*_max_ and AUC_0–24 h_) after the administration of the 4 mg dose to infants of 1 to 3 months were higher and more variable than for older infants (3 to 24 months) (16,17,19). The higher systemic exposure in the younger subgroup when given the dose of montelukast was attributed to their smaller body weight, and to the levels of CYP3A4, which are only about 30 to 40% of adult levels in ages younger than 3 to 12 months (17).

**Fig. 3:**
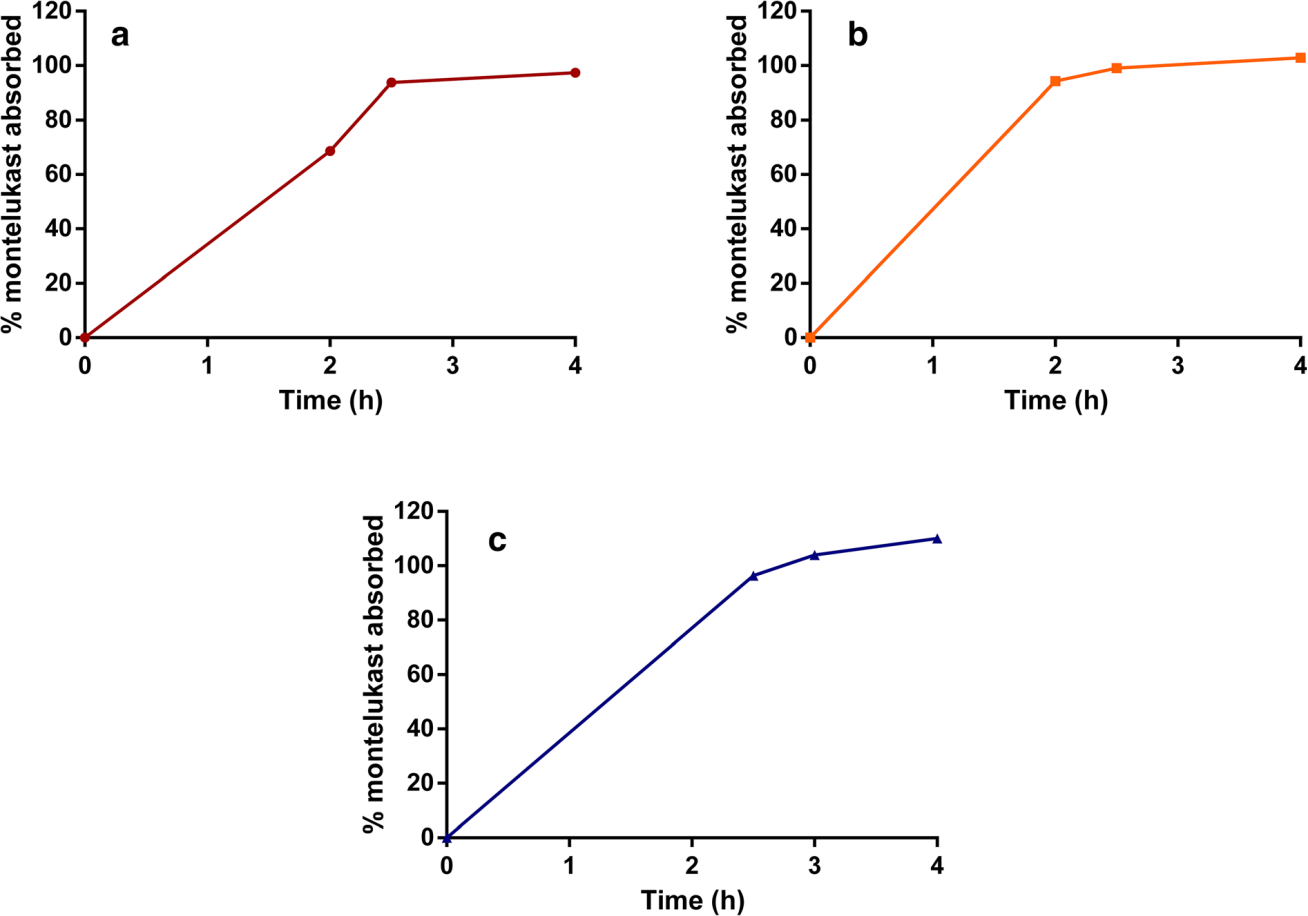
% montelukast absorbed *in vivo* after administration of Singulair^®^ granules (4 mg) to **a** 1 to 3 months infants with formula (17); **b** 3 to 6 months infants with applesauce (16); and **c** 6 to 12 months infants with applesauce (19). The % absorbed was calculated with the Wagner-Nelson equation

The *in vivo* % absorbed profiles of montelukast after administration of Singulair^®^ granules, in the different subgroups of infants (calculated with deconvolution of the plasma profiles after oral administration using the Wagner-Nelson equation (16,17,19,23)), are shown in Fig. 3.

### *In Vitro*-*In Vivo* Relationships for Singulair^®^ Granules

**Fig. 4:**
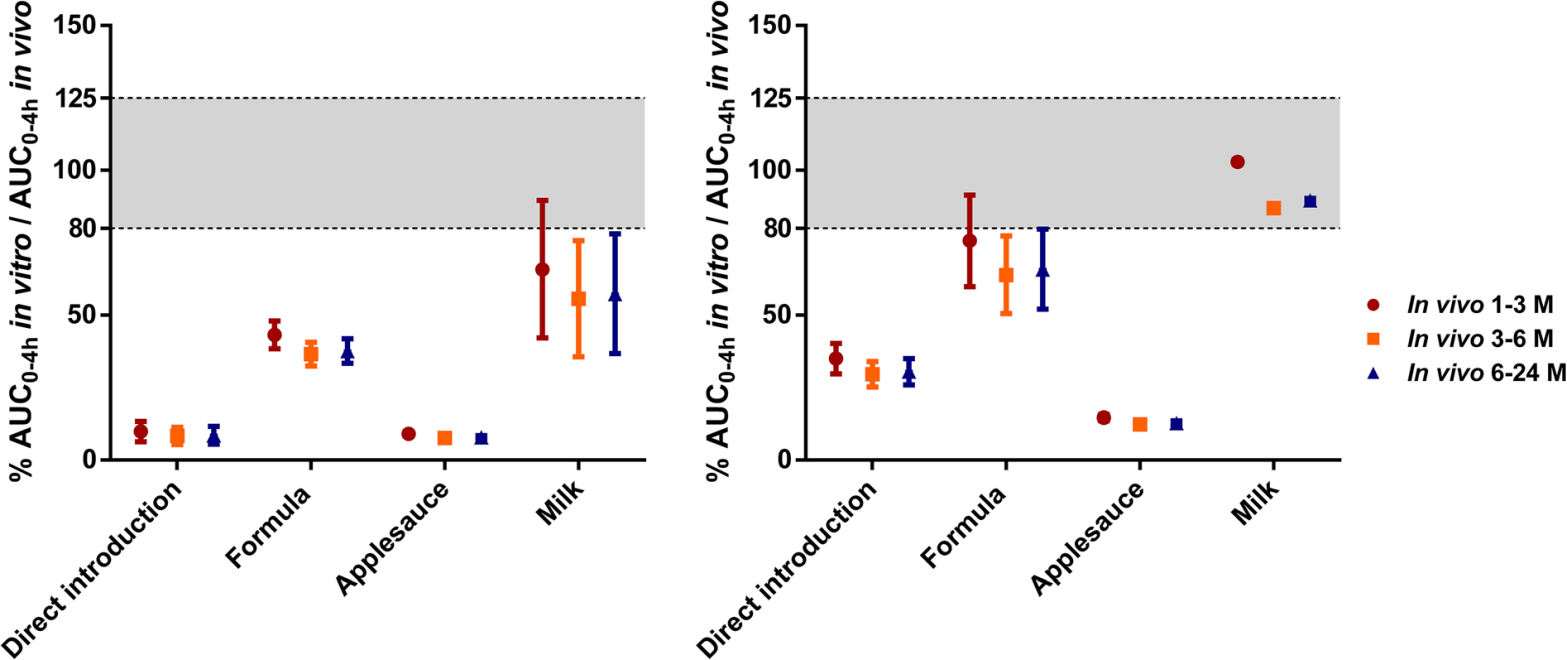
Ratio (%) between each AUC_0–4 h_ *in vitro* and AUC_0–4 h_ *in vivo* (AUC_0–4 h_ *in vitro*/AUC_0–4 h_ *in vivo* × 100). AUC_0–4 h_ *in vitro*: **a** *in vitro* fasted-state gastric followed by fasted-state intestinal conditions; and **b** *in vitro* fasted-state gastric followed by fed-state intestinal conditions. AUC_0–4 h_ *in vivo*: calculated from the absorption profiles after deconvolution of the *in vivo* plasma profiles of Singulair^®^ granules (4 mg) in infants (16,17,19); (grey area represents the range criteria (80–125%) set for prediction of *in vivo* drug performance)

The ratios between the AUC_0–4 h_ *in vitro* (from the *in vitro* dissolution profiles of Singulair^®^ granules directly administered or mixed with milk, formula or applesauce, under fasted-state gastric conditions followed by fasted- or fed-state intestinal conditions) and the AUC_0–4 h_ *in vivo* (from the absorption profiles in infants 1 to 3 months, 3 to 6 months and 6 to 24 months) are presented in Fig. 4. In the cases of direct introduction of Singulair^®^ granules and mixing with applesauce, the *in vitro* drug dissolution was much slower than the *in vivo* absorption of montelukast, in all subgroups (% AUC_0–4 h_ *in vitro*/AUC_0–4 h_ *in vivo* ratio lower than 80%).

For the mixing of Singulair^®^ granules with formula scenario, when testing under fasted-state gastric conditions followed by fed-state intestinal conditions, the AUC_0–4 h_ *in vitro*/AUC_0–4 h_ *in vivo* ratio fell inside the 80–125% limits for infants of 1 to 3 months old. The *in vitro*-*in vivo* ratio was lower than the 80% limit for all subgroups when testing under fasted-state gastric conditions followed by fasted-state intestinal conditions. These results indicate that the biorelevant *in vitro* dissolution test under fasted-state gastric conditions followed by fed-state intestinal conditions gives a good prediction of the *in vivo* product performance for the 1 to 3 months subgroup, when the granules are mixed with formula. For the Singulair^®^ granules mixed with milk, when testing under fasted-state gastric conditions followed by fed-state intestinal conditions and comparing with *in vivo* results in infants from all subgroups, the AUC_0–4 h_ *in vitro*/AUC_0–4 h_ *in vivo* ratio fell inside the 80–125% limits. For infants of 1 to 3 months, a good relationship was also found between the *in vivo* performance and the *in vitro* performance when using milk and testing under fasted-state gastric conditions followed by fasted-state intestinal conditions.

Results from this study suggest that the *in vivo* studies were likely performed in the fed state (as the prandial state of the infants is not evident) or that the practice of medicine co-administration with food and drinks might trigger fed-state conditions *in vivo*. The biorelevant *in vitro* dissolution test (fasted-state gastric conditions followed by fed-state intestinal conditions) using milk gives the best prediction of the *in vivo* product performance for infants of all subgroups. This is likely related to the high solubility of montelukast in this vehicle and its carbohydrate, protein and fat content which is similar to that observed in the stomach after administration of meals. Further investigations would be helpful to confirm and further optimise the dissolution testing parameters for a predictive, physiologically relevant methodology (1,2). A detailed characterisation of paediatric GI contents of different age groups *in vivo* would be valuable for further development of these paediatric biopharmaceutical methods.

## CONCLUSIONS

The practice of mixing medicines with food and drinks may affect drug behaviour, leading to potential clinical implications. As emphasised in the recent FDA draft guidance on the use of vehicles for drug administration, this potential impact should be assessed during formulation development/evaluation by using different biopharmaceutical tools. In this study, a predictive biorelevant dissolution test was developed to investigate the impact of medicine co-administration with soft food and drinks on the dissolution performance of montelukast, a poorly soluble compound.

Dissolution of montelukast was significantly affected after mixing the tested formulation with vehicles compared with the drug dissolution after direct administration of the formulation. Moreover, drug dissolution was significantly higher when testing under fed-state intestinal conditions in comparison with the fasted state.

The biorelevant *in vitro* dissolution test (fasted-state gastric conditions followed by fed-state intestinal conditions) of the Singulair^®^ granules mixed with milk scenario led to the best prediction of the *in vivo* drug performance in infants of all subgroups (1 to 3 months, 3 to 6 months and 6 to 24 months). Results from this study suggest that the *in vivo* studies were probably performed in the fed state or that the practice of medicine co-administration with food and drinks might have triggered fed-state conditions *in vivo*.

The good relationship between the *in vitro* drug dissolution and *in vivo* absorption obtained in this study when the granules were mixed with milk demonstrates the potential utility of biorelevant *in vitro* dissolution testing to understand the potential impact of co-administration of paediatric medicines with vehicles on drug performance and avoid potential clinical implications.
